# Workplace violence against nurse: a systematic review and meta-analysis in Ethiopia

**DOI:** 10.1186/s12912-025-03243-1

**Published:** 2025-05-26

**Authors:** Sadik Abdulwehab, Frezer Kedir

**Affiliations:** 1 School of Nursing, College of Health Science, Wollega University, P.O. Box: 395, Nekemte, Oromia Ethiopia; 2 School of Nursing, Jimma University, Southwest Oromia, Ethiopia

**Keywords:** Workplace violence, Nurses, Ethiopia

## Abstract

**Background:**

Workplace violence is a global public health concern, especially in developing nations. Nurses, due to the nature of their professional duties, often face an elevated risk of workplace violence. This risk is further exacerbated by the lack of adequate safety precautions and the presence of multiple potential perpetrators, making the work environment particularly hazardous for them.

**Objectives:**

This systematic review explores workplace violence against nurses in Ethiopia, its prevalence, causes, Perpetrators of violence in the Nurses’ profession, consequences, influence on the profession, and strategies to prevent incidents based on relevant literature review.

**Review method and data sources:**

The study sourced evidence from electronic databases like PubMed, Science Direct Scopus, Web of Science, and Google Scholar till January 30, 2024. The data was extracted from February 01–10 and later analyzed from February 15–March 15, and the report generation from March 15 to April 05, 2024, and reported findings per Preferred Reporting Items for Systematic Reviews and Meta-Analyses extension for Scoping Reviews. The study used pooled odds ratio and pooled proportion to assess exposure and outcomes. A meta-analysis was conducted using compressive met-analysis V4 packages, with forest plots for visual representation. Heterogeneity was evaluated using Cochran’s Q-test and I² statistic. Sensitivity analysis was performed by systematically excluding one study.

**Result:**

In Ethiopia, workplace violence against nurses was a significant issue, with an incidence ranging from 26.7 to 64%. The most common form of violence was verbal abuse, followed by physical, psychological, and sexual harassment. Factors like age, gender, marital status, job type, work environment, and staffing levels contribute to the issue. Over half of nurses change shifts due to concerns about violence, community dissatisfaction, unsatisfactory treatment, and challenging nurse-patient relationships.

**Conclusion:**

The prevalence of WPV among nurses in Ethiopia is increasing. Nurses face a high risk of workplace violence due to direct patient contact and the nature of the profession. Thus, healthcare organizations can work towards creating a safer and more supportive environment for nurses, enforce a zero-tolerance policy, improve patient-nurse relationships, and provide psychological support to ultimately reduce the prevalence of workplace violence and ensure the well-being of healthcare professionals.

**Prospero registration number:**

CRD42024601317.

## What is already known

- Workplace violence against nurses in Ethiopia is prevalent and increasing.
- Nurses are at high risk due to the nature of their profession and direct patient contact.
- Multiple perpetrators contribute to workplace violence, including patients, visitors, and staff members.

## What this paper adds

• The pooled prevalence of workplace violence among nurses in Ethiopia is approximately 39.61%, with rates increasing over time.
• Verbal abuse is the most frequently reported form of workplace violence, followed by physical, psychological, and sexual harassment.
• Healthcare organizations need to establish violence prevention strategies, enforce zero-tolerance policies, and improve patient-nurse relationships to reduce violence and ensure nurses’ safety.

## Introduction

The World Health Organization (WHO) defines workplace violence (WPV) as incidents involving work-related abuse, threats, or assaults including physical, sexual, verbal, and psychological abuse, and harassment targeting health workers [1]. WPV has emerged as a pressing global public health issue, particularly affecting healthcare professionals in developing countries with under-resourced health systems [2]. Nurses are among the most frequent victims, as violence in healthcare settings has reached alarming proportions globally [3].

Studies show that a significant proportion of WPV occurs within the healthcare sector, with approximately 88% of healthcare professionals in developing nations reporting exposure to various forms of violence [4, 5]. Despite its widespread occurrence, violence against nurses often remains overlooked and underreported, contributing to its persistent impact on the profession [6].

WPV includes verbal abuse, physical assault, and sexual harassment, all of which have detrimental effects on both healthcare workers and organizational performance [7–10]. The consequences extend beyond individual harm and include increased legal costs, compensation claims, staff turnover, loss of productivity, and the need for heightened security measures [10, 11].

In Ethiopia, nurses face escalating challenges related to WPV, contributing to job dissatisfaction, low morale, and high turnover rates [1, 6, 8, 12]. However, reporting remains limited due to fears of retaliation, a lack of institutional support, and a general perception that reporting will not lead to meaningful change [3, 13, 14]. Evidence also suggests that the experience of violence varies by gender and age, with younger and female nurses being disproportionately affected by verbal, sexual, and psychological abuse [15].

Broader systemic factors such as inflation, financial pressure on health institutions, staff shortages, and burnout may further exacerbate workplace tensions [16]. Additionally, the increasing emphasis on patients’ rights to participate in decision-making, though beneficial, can sometimes contribute to misunderstandings and workplace conflict [16].

While the Ethiopian Labor Proclamation No. 377/2003 includes occupational safety guidelines, the absence of a comprehensive national strategy for WPV management remains a critical gap [17]. Reports show high levels of workplace injuries and service inadequacies, despite favorable policy intentions [18]. Although international organizations like the Joint Commission and the US Occupational Safety and Health Administration have established WPV prevention guidelines, violence hinders job satisfaction and performance [12, 19].

Workplace violence against nurses in Ethiopia is a significant concern. However, existing research has largely focused on general occupational hazards rather than the specific forms and effects of violence against nurses [8, 15, 20, 21]. As a result, the true extent, patterns, and consequences of WPV in the Ethiopian nursing context remain poorly understood. This systematic review aims to fill this gap by synthesizing available evidence on the prevalence, trends, and impacts of WPV on nurses in Ethiopia. It also highlights underreporting issues and explores factors that hinder effective responses. Ultimately, the review provides a basis for evidence-informed policy development and workplace interventions aimed at protecting Ethiopian nurses and enhancing healthcare system safety.

## Methods

### Aim of the review

This systematic review aimed to assess the prevalence, impact, and contributing factors of workplace violence (WPV) against nurses in Ethiopia and to identify potential strategies for prevention.

### Design

This review followed the Preferred Reporting Items for Systematic Reviews and Meta-Analyses (PRISMA 2020) checklist guidelines [22]. A comprehensive survey of studies on WPV targeting nurses was conducted to evaluate the methods employed by researchers in this field.

### Research question

This review was guided by the following question: What are the pooled prevalence, impact, and contributing factors of workplace violence against nurses in Ethiopia, and what strategies have been proposed to mitigate future occurrences? The research question was aligned with the review’s objectives, title, and the selected articles.

### Inclusion and exclusion criteria

**Fig. 1:**
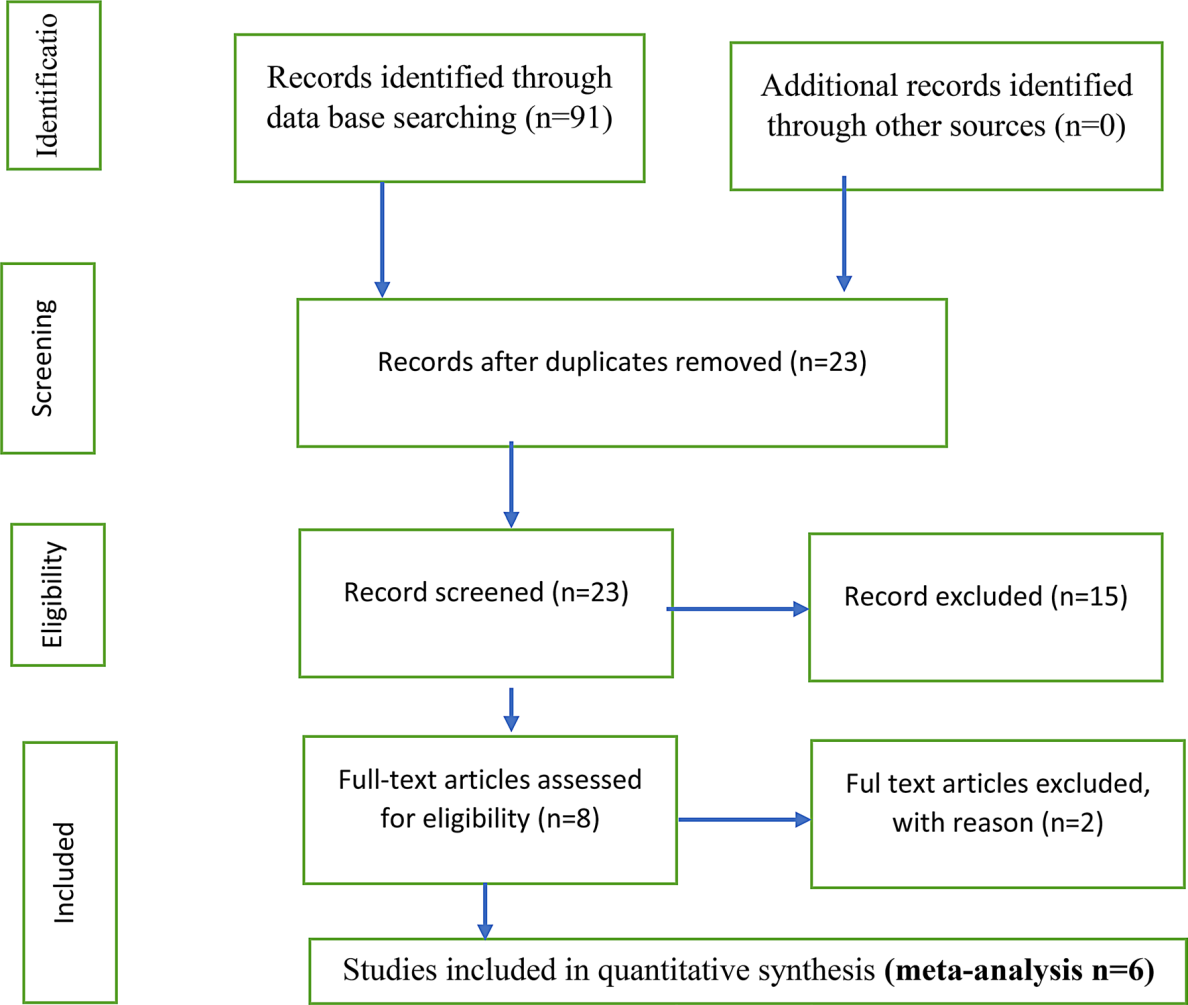
Flow chart of the study selection process

Studies were included if they focused on workplace violence against nurses in Ethiopia, regardless of the year of publication. Studies addressing violence against healthcare providers other than nurses were excluded. The selection process is detailed in Fig. 1.

### Search strategy

A comprehensive literature search was conducted using the following electronic databases: PubMed, Science Direct, Scopus, Web of Science, and Google Scholar. The search employed individual and combined Medical Subject Headings (MeSH) terms such as verbal abuse, aggressiveness, nurses, assault, physical violence, psychological violence, Ethiopia, and workplace violence. The database search covered all publications up to January 30, 2024, and updates continued until submission. Data extraction occurred from February 1–10, 2024, with analysis conducted between February 15–March 15, and final report generation from March 15–April 5, 2024. This review was registered with PROSPERO under registration number CRD42024601317.

### Search outcomes

The initial search yielded 101 articles across the databases (PubMed = 24, CINAHL = 14, Scopus = 26, Web of Science = 18, Google Scholar = 19). Articles were exported to Excel and managed using Zotero. After duplicate removal and applying inclusion criteria, 23 articles were screened, with 8 evaluated for full-text eligibility. Of these, 6 articles met the inclusion criteria.

Titles and abstracts were independently reviewed by two authors. Any discrepancies in eligibility assessment were resolved through discussion. The selection process is illustrated in Fig. 1.

### Data extraction

Data were extracted using a structured template based on the Joanna Briggs Institute (JBI) methodology [23]. Extracted data included: first author, publication year, country, study objectives, concepts, design, setting, sample size, sampling method, data collection techniques, key findings, recommendations, and limitations. Two reviewers independently extracted the data, and disagreements were resolved through discussion.

### Data synthesis and reporting

Extracted data were organized in Excel and analyzed using descriptive statistics (frequency, percentages, means, and ranges). Results were presented using descriptive summaries, tables, and figures. Reporting was guided by the PRISMA-ScR checklist (2020 version) [22]. All extracted data relevant to the research question are summarized in Table 1.

**Table 1:** Detail of all included articles on WPV against nurse in Ethiopia,2024

Author, publication year (year of actual data collection)	Study aim/objective	Study design & setting	Participants, sample size & sampling method	Data collection method	Key findings	Recommendation	Limitation
Bekalu, Yemane Eshetu; Wudu, Muluken Amare,2022	To evaluate the extent and contributing variables of workplace violence among public hospital nurses.	A cross-sectional study carried out at 14 government hospitals in a clinical context.	568 nurses were included using the census	Using PubMed, Google Scholar, Scopus, Web science.	**Prevalence** of WPV over past 12onths = 56% **Prevalence** by Gender: (60.9%) female, male (39.1%), by age: 30yrs. (54.6%), 35yrs (37.8%). **Types**: 21% physical assault,49% verbal abuse,17.2% claimed bullying, and 7.5% sexual harassment **Place Where the Violence Takes**: around 80% of the occurred inside the hospital. **Correlates**: Female nurses(*p* < 0.001), Age > 40 (*p* = 0.018), nurses those alcohol users(*p* < 0.001), nurses who had contact with male patients (*p* < 0.001) were more likely to experience than counterparts. **Perpetrators**: patients and patients’ relatives (90%), Staff supervisors (25%) **Policy on violence**: non-existent **Cause of Violence**: Patient waiting time, unmet patients’ needs.	Requires significant community-and facility-based behavioral change. It is important to conduct health promotion initiatives on workplace violence, with a special emphasis on patients and nurses.	findings conducted solely in English, with inadequate research methodologies, and with subjective evaluations of the findings. Recall bias could result from this.
Fute, Mathewos; Mengesha, Zelalem Birhanu; Wakgari, Negash; Tessema, Gizachew Assefa,2014	To determine the frequency of workplace violence among nurses employed by healthcare facilities and the factors that contribute to it.	A clinical cross-sectional investigation carried out in two governmental hospitals and ten health facilities	660 nurses were included using the census	Using PubMed, Google Scholar, Scopus, Web science.	**Prevalence** of WPV over past 12onths = 29.9% **Prevalence** by Gender: 75.5% female, male 24.5%, by age: below 25. (42,2%), 35yrs (36.4%),19.4% above 36 years old. by experience: or 5yrs (67.6%) **Types**: Verbal abuse 89.6%, physical violence 18.8%, and sexual harassment 13%. **Place Where the Violence Takes**: around 80% of the occurred inside the hospital. **Correlates**: Being female, having younger age, having short work experience, and having assignments in emergency and inpatient departments were associated with workplace violence. **Perpetrators**: patients and patients’ relatives (45%), **Policy on violence**: non-existent **Cause of Violence**: Patient waiting time, nature of clinical setting, and working in the night shift.	Health care facilities ought to set up health and safety initiatives to handle and avoid workplace violence. Strategies for preventing workplace violence should be the focus of policymakers and stakeholders.	findings conducted solely in English, with inadequate research methodologies, and with subjective evaluations of the findings. could result in prejudice in recollections. Lack of a p-value report.
Legesse, Henok; Assefa, Nega; Tesfaye, Dejene; Birhanu, Simon; Tesi, Seid; Wondimneh, Fenta; Semahegn, Agumasie,2021	the severity of workplace violence against nurses and the factors that contribute to it among nurses	A cross-sectional investigation carried out in six government hospitals in a clinical context	603 nurses were included using the census	Using database on PubMed, Google Scholar, Scopus, Web science.	**Prevalence** of WPV over past 12onths = 64.0% **Prevalence** by age: below 35 (66.3%), above 35yrs (33.7%), by educational level: diploma 11.1%, degree and above 88.9%, My experience: below 5 years 18.2%, 5 years up to 10 years 24.5%, above 10 years and 21.2%, by shift: those done in night shift 56.2%, By department: specialized unit 14%, Gyn/Obs 11%, medical ward 8%, Ambulatory/OPD 7.8%. **Types**: Verbal abuse 61%, physical violence 30.2%, bullying/mobbing 22.1%, and sexual harassment 12.4%. **Place Where the Violence Takes**: around 64% of the occurred inside the hospital. **Correlates**: Department/working unit in medical, surgical, psychiatry, and emergency unit, being worried about violence, witnessing physical violence, reporting procedures, and institution policy on WPV was associated. **Perpetrators**: patients and patient’s relatives **Policy on violence**: non-existent **Cause of Violence**: Patient waiting time, nature of clinical setting, and working in the night shift.	Stakeholders could collaborate on managing violent situations and early risk identification. To stop workplace violence against nursing professionals, provide methods for reporting and sanctioning violent incidents while utilizing contextual tactics.	findings conducted solely in English, with inadequate research methodologies, and with subjective evaluations of the findings. Recall bias and social desirability may result from this. not examined the questionnaire’s psychometric properties. A study carried out in a restricted location.
Tiruneh, Bewket Tadesse; Bifftu, Berhanu Boru; Tumebo, Akililu Azazh; Kelkay, Mengistu Mekonnen; Anlay, Degefaye Zelalem; Dachew, Berihun Assefa,2015	To evaluate the frequency of violence at work and related variables among nurses	A cross-sectional study carried out in three public referral hospitals, a clinical environment	386 nurses were included using the census	Searching in PubMed, Google Scholar, Scopus, Web science	**Prevalence** of WPV over past 12onths = 26.7% **Prevalence** By gender: high prevalence among males 59.2%, and females 40.8%. By degree level: First-degree holder nurses were the most affected group 78.64%. My experience: 1–5 years 39.8%, < 1 year 30.1%. by shift: majority during night 52.9%. **Types**: physical violence 60.2%, psychological violence 39.8% **Place Where the Violence Takes**: around 64% of the occurred inside the hospital. **Correlates**: being age 18–39 (*p* = 0.034), a small number of staff in the same shift means < 5 (*p* = 0.02), working in the male ward (*p* = 0.001), history of workplace violence (*p* = 0.002), being single (*p* = 0.001) and separated/ widowed (*p* = 0.003) were suggestive of being independent predictors of workplace violence. **Perpetrators**: Majority by Relatives of the patients (60.2%), by staff (27.2%), by patients (11.7%), and by Manager (1%). **Policy on violence**: non-existent **Cause of Violence**: Patient waiting time, nature of the clinical setting, working in the night shifts, and a small number of staff in the same shift.	collaborating with the hospital administration, staff nurses, and nursing association representatives to develop a preventative plan.	findings conducted solely in English, with inadequate research methodologies, and with subjective evaluations of the findings. Recall bias could result from this. Small sample size, incorrect interpretation or narration of the tables. Just two forms of violence—physical and psychological—should be the focus.
Weldehawaryat, Haymanot Nigussie; Weldehawariat, Feleke Gebremeskel; Negash, Firdawek Getahun,2018.	To evaluate the frequency of violence at work and related variables among nurses	A cross-sectional study carried out in 19 public health facilities in a clinical setting	354 nurses were included using the census	Using PubMed, Google Scholar, Scopus, Web science.	**Prevalence** of WPV over past 12onths = 43.1% **Prevalence** By Age: in years 18–30years age category 68.7%, by gender: being female 60.7%, by marital status: Live with spouse 56.7%, by educational level: Have no bachelor’s degree 68.0%, by place of work: hospital 70.0%, health center 30.0%, by experience: <10 years 74.0%, by department unit: Gyn/Obs 23.3%, OPD 20.7%, Pediatrics 20.0%, Surgical 14.0%, Emergency 11.3%, lastly in medical ward 8.7%. by shift: during the night shift 88.0%. **Types**: verbal abuse 28.2%, physical violence 13.5%, 10.3% were bullied/mobbed, and 7.2% faced sexual harassment at least once in the last 12 months. **Place Where the Violence Takes**: around 87.2% of the occurred inside the hospital. **Correlates**: Female nurses (*p* = 0.007), those who live without a spouse (*p* = 0.006), those who drink alcohol (*p* = 0.04), and those who chew chat (*p* = 0.016) were more likely to suffer from workplace violence in public health facilities. **Perpetrators**: Relatives of patient/client 55.3%, Patient/client 25.5%, external colleague/ worker 4.3%, Supervisor 8.5%, Staff member 4.3%. **Policy on violence**: non-existent **Cause of Violence**: Patient waiting time, nature of clinical setting (high stress full), working in the night shifts, and using chat and alcohol	Lawmakers and other interested parties pay close attention to instances of workplace violence and set up a systematic procedure for reporting incidents of violence.	findings conducted solely in English, with inadequate research methodologies, and with subjective evaluations of the findings. Recall bias could result from this. Perpetrator factors, such as the offenders, are not included in the study. tiny sample size.
Yenealem, Dawit Getachew; Mengistu, Avier Mesfin,2017	To investigate instances of physical aggression and related issues among nurses	A cross-sectional study carried out in nine public health facilities in a clinical setting.	339 nurses were included using the census	Using PubMed, Google Scholar, Scopus, Web science.	**Prevalence** of WPV over past 12onths = 28.9% **Prevalence** by department unit: Inpatient departments 49.6%, Emergency departments 12.1%, OPD-out patient department 32.7%.by staff number per shift: a small number of staff (1–5) per shift 32.2%. by experience: less than five years 64.6%. by educational level: degree and above 89.7% and diploma 10.3%. **Types**: conduct physical violence (42.3%) only. **Place Where the Violence Takes**: around 75.8% of the occurred inside the hospital. **Correlates**: Exposure to verbal abuse(*p* < 0.05), working in an emergency(*p* < 0.05) and inpatient departments(*p* < 0.05) and perceived level of concern(*p* < 0.05) are the precursors of experiencing physical violence. **Perpetrators**: Relatives of patient/client and patient/client. **Policy on violence**: non-existent **Cause of Violence**: Patient waiting time, nature of the clinical setting, working in the night shifts and unit of the department, a small number of staff during the shift and using chat and alcohol, unconducive environment (fear of having safety during war/conflict)	spending time and money on training that includes de-escalation techniques for nurses, restricting them, making hospitals more welcoming, and developing standard operating procedures for reporting and managing violence	concentrating on a specific aspect of violence, utilizing a weak study design, conducting research solely in the English language, and making subjective assessments of the research. Recall bias could result from this. little sample size. omit the real p-value. The relationship is only physical. Physical violence and the idea of WPV were not isolated from each other.

### Quality appraisal

A critical appraisal of the included studies was conducted using the AXIS tool for cross-sectional studies [24]. The checklist assessed aspects such as clarity of purpose, methodology, sample recruitment, ethical considerations, data analysis, findings, and local relevance.

Six studies were appraised, each evaluated on nine criteria related to methodological rigor, bias, and applicability. Studies scoring **≥ 7** were considered high-quality and included in the final analysis. Quality appraisal results are summarized in Table 2.

**Table 2:** Critical appraisal results of eligible studies in this study on workplace violence against the nursing profession in Ethiopia, 2024 (*n* = 6)

Checklist	Article					
	Bekalu et al.	Fute et al.	Legesse et al.	Tiruneh et al.	Weldehawaryat et al.	Yenealem et al.
Was the sample frame appropriate to address the target population?	Y	Y	Y	Y	Y	Y
Were study participants sampled appropriately?	Y	Y	Y	Y	Y	Y
Was the sample size adequate?	Y	Y	Y	Y	Y	N
Were the study subjects and the setting described in detail?	Y	Y	Y	Y		Y
Was the data analysis conducted with sufficient coverage of the identified sample	Y	Y	Y	Y	Y	Y
Were the valid methods used for the identification of the condition?	Y	Y	Y	Y	Y	Y
Was the condition measured in a standard, reliable way for all participants?	Y	Y	Y	Y	Y	Y
Was there appropriate statistical analysis?	Y	Y	Y	Y	Y	Y
Was the response rate adequate, and if not, was the low response rate managed appropriately	Y	Y	Y	Y	Y	Y
**Total score of each article**	**9**	**9**	**9**	**9**	**9**	**8**

### Statistical analysis

Pooled odds ratios with 95% confidence intervals (CIs) were calculated to assess associations between exposures and outcomes. The pooled proportion was used to summarize prevalence data across studies. Meta-analysis was performed using Comprehensive Meta-Analysis (CMA) Version 4 software. Forest plots were generated to visualize pooled effect sizes.

Heterogeneity was assessed using Cochran’s Q-test and quantified with the I² statistic; an I² value > 50% indicated substantial heterogeneity. Funnel plots and Egger’s regression test were used to assess publication bias. Sensitivity analysis was conducted by sequentially removing individual studies to test the robustness of the pooled results. A random-effects model was used due to expected variability across studies.

## Result

### Characteristics of the reviewed studies

Six original research papers on workplace violence among Ethiopian nursing professionals were included in this evaluation after they satisfied the inclusion requirements (See Table 2). All of them were cross-sectional study designs [7, 15, 20, 21, 25, 26]. Two studies were excluded because one study focused solely on female nurses and one study lacked full-text availability [6, 27].

### Prevalence (12 months) of workplace violence across the study

**Fig. 2:**
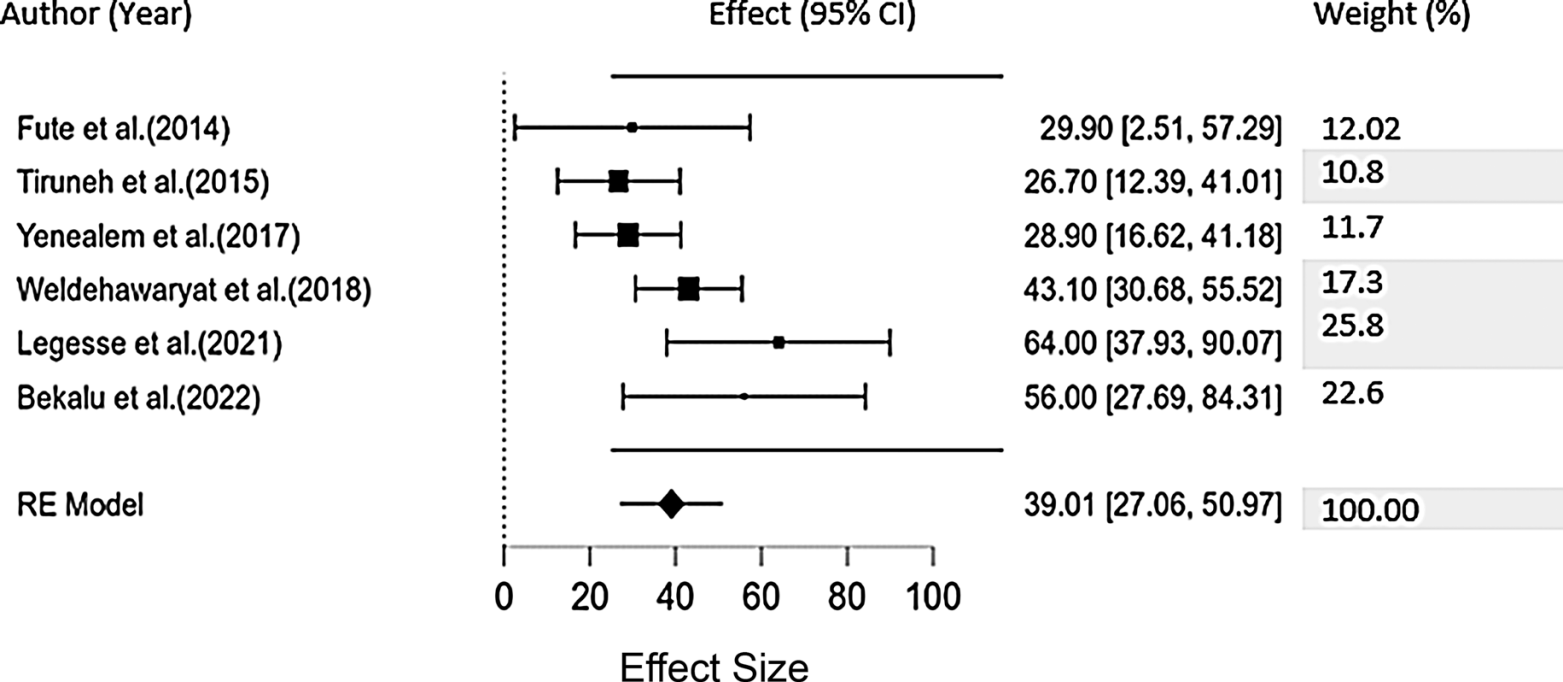
Forest plot showing the pooled prevalence of workplace violence against the nurses in Ethiopia (*n* = 6)

Different regions of Ethiopia were reported to have varying rates of workplace violence throughout the literature. All of them were conducted in clinical areas in public institutions with variance in participants included and there is a difference in prevalence rate across all articles. In public health institutions in Southern Ethiopia, Fute et al. [15] found that the prevalence of workplace violence against nurses was 29.9%, after a year study done by Tiruneh et al. [20] in Northwest Ethiopia, which accounts for 26.7%. Again, after two years similar study done by Yenealem et al. [26] in Northwest Ethiopia yielded 28.9%. Again, a study done by Weldehawaryat et al. [25] in southern Ethiopia accounts for 43.1%, and a study done by Legesse et al. [21] in eastern Ethiopia, accounts for 64.0%. Later, Bekalu et al. [7] conducted a study in Northeastern Ethiopia that showed 56% (Table 3). The Pooled prevalence of workplace violence against a nurse in Ethiopia was 39.61% and this ranged from 36.52 to 42.70% (Fig. 2) with low heterogeneity tests (I^2^ = 58.4) and no significant publication bias (A small p-value = 0.544 from Egger’s test).

**Table 3:** List of articles done in Ethiopia and its 6–12-month prevalence of workplace violence against the nursing profession

First author	Year	Prevalence (95% CI)	Sample	Cases
Bekalu et al. (2022)	2022	56% (51.8–60.2)	568	299
Fute et al. (2014)	2014	29.9(26.5–33.5)	660	197
Legesse et al. (2021)	2021	64.0% (60.2–67.7)	603	386
Tiruneh et al. (2015)	2015	26.7% (40.6–75.8)	386	103
Weldehawaryat et al. (2018)	2018	43.1% (37.9–48.6)	354	153
Yenealem et al. (2017)	2017	28.9% (24.8, 33.9)	339	98

Out of the 6 articles, only four specified gender differences in the prevalence of workplace violence among nurses [7, 15, 20, 25, 26]. Reports indicated that 60.9% of females [7], 75.5% of females [15], 40.8% of females [20], and 60.7% of females report presence of workplace violence [25].

Out of the 6 articles, only four specified age differences in the prevalence of workplace violence among nurses. The prevalence of workplace violence was notably high across various age groups, with those below 30 years old with a prevalence of 54.6% [7], 66.3% [21], and 68.7% [25], and those below 25 account for 42.2% [15].

Out of 6 articles five articles specified level of work experience differences in the prevalence of workplace violence among nurses. There is a high prevalent rate of workplace violence among those 5 and above years of experience 67.6% [15], below 5 years 18.2%, 5 years up to 10 years 24.5%, above 10 years and 21.2% [21], 1–5 years 39.8%, <1year 30.1% [20], below 10 years 74.0% [25], less than five years 64.6% [26]. Out of 6 articles four articles specified differences in educational level on the prevalence of workplace violence among nurses. There Is a high prevalence of workplace violence among those has a degree and above 88.9% [21], First-degree holder nurses 78.64% [20], Have no bachelor’s degree 68.0% [25], degree and above 89.7% [26].

Out of 6 articles three articles specified differences across department units on the prevalence of workplace violence among nurses. That nurse works in a specialized unit 14%, Gyn/Obs 11%, medical ward 8%, Ambulatory/OPD 7.8% [21], Gyn/Obs 23.3%, OPD 20.7%, Pediatrics 20.0%, Surgical 14.0%, Emergency 11.3%, and medical ward 8.7% [25], Inpatient departments 49.6%, Emergency departments 12.1%, OPD-out patient department 32.7% [26]. Out of 6 articles three articles, specified differences across work shifts on the prevalence of workplace violence among nurses. The nurse works on the night shift 56.2% [21], the majority during the night 52.9% [20], during the night shift 88.0% [25].

The incidence of workplace violence among nurses varies across different types of work facilities. According to a study by Weldehawaryat et al. [25], it accounts for 70.0% of all cases. Out of 6 articles only one [25] specified differences in the prevalence of workplace violence among nurses as per the number of nurses per shift (sample size of nurses in shift) as 1–5 nurses per shift (21.2%),6–10 nurses per shift (5.0%), above 10 nurses per shift (2.7%).

### Sensitivity analysis

Sensitivity analysis was conducted to examine the robustness of the pooled effect size. Each study was systematically removed, and the meta-analysis was re-run for the remaining studies. The pooled odds ratio ranged between 36.66 (95% CI: 25.7–47.6) and 44.36 (95% CI: 30.53–58.19) when individual studies were excluded. Heterogeneity (I²) showed minor changes across iterations, ranging from 54 to 58.4%. The results suggest that the meta-analysis findings are robust and not overly influenced by any single study.

**Fig. 3:**
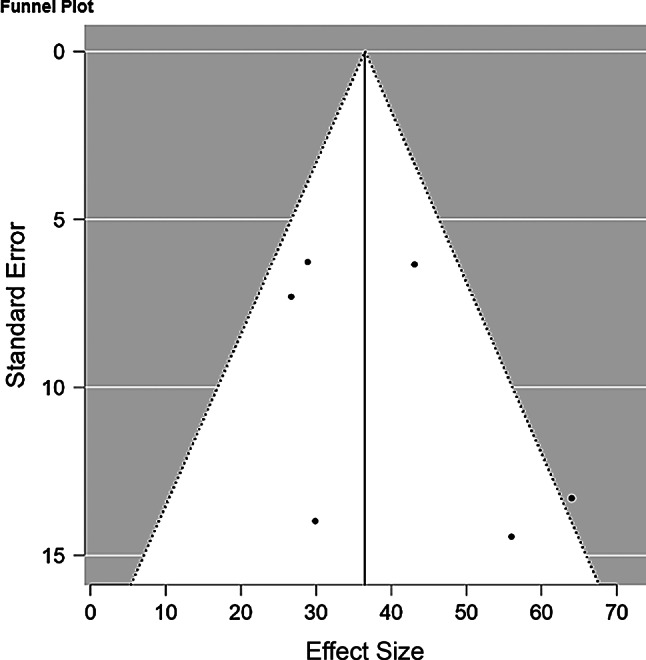
Funnel plot with pseudo 95% confidence limits among workplace violence against the nurses

A funnel plot with pseudo 95% confidence limits was utilized to assess potential publication bias among the included studies and it shows a symmetric, inverted funnel shape, indicating that studies are distributed evenly around the average effect size. In this review, the funnel plot’s symmetry was evaluated to determine the presence of publication bias, which is crucial for ensuring the validity and reliability of the systematic review’s conclusions regarding workplace violence against nurses (Fig. 3).

### Types of workplace violence among nurses profession

There have been differences in the rates of physical, verbal, emotional, psychological, and sexual forms of workplace violence reported in the literature [7, 15, 20, 21, 25, 26]. Physical forms of workplace violence were ranged from 18.8- 60.2% [7, 15, 20, 21, 25, 26]. From the category of physical violence: verbal form workplace violence ranged from 28.2-89.6% [7, 15, 21, 25], and claimed bullying/mobbing ranged from 10.3 to 22.1% [8, 21, 22]. Tiruneh et al. show the prevalence of psychological violence is 39.8% [20]. Sexual harassment a form of workplace violence ranged from 7.2 to 13% [7, 15, 21, 25].

### Perpetrators of violence against nurses’ profession

The research reviewed showed that, in varying proportions, patients’ and clients’ relatives, patients’ and clients’ visitors, staff members (such as senior nurses, doctors, and other professionals), outside coworkers, managers, and supervisors were the people who abused nurses. Relatives of patients or clients made up 45–90% [7, 15, 20, 21, 25, 26]. The patients/clients as perpetrators of workplace violence were reported at a range of 11.7-90% [7, 15, 20, 21, 25, 26]. By staff members ranged from 4.3-27.2% [20, 25], by supervisors ranged from 8.55 -25% [7, 25], 1% by the manager [20], and external colleague/ worker 4.3% [25].

### Correlates of violence among nurses profession

There is High exposure to WPV both at a younger age and older age [7, 15, 20]; being female nurses was associated with higher exposure to workplace violence [7, 15, 25], Nurses who consumed alcohol [7, 25] or chewed khat [25] were more likely to experience workplace violence [7, 20].

Regarding work experience Nurses with less work experience were more likely to be exposed to workplace violence [15, 22]. Department/unit of work also influenced workplace violence, as nurses who work in the emergency and inpatient departments [15, 21, 26], male ward [20, 21], surgical ward, and psychiatry ward [21] were involved in experiencing workplace violence.

Nurses working with a small number of staff during the same shift [20], those who are single, separated, or widowed, or live without a spouse [20, 25], as well as those concerned about violence, who have witnessed physical violence, and who are familiar with reporting procedures and institutional policies on workplace violence [21], were identified as independent predictors of workplace violence. Additionally, exposure to verbal abuse, a history of workplace violence, and the level of concern were also found to be significant predictors [20, 26], as detailed in Table 1.

### Causes of workplace violence

All the reviewed articles indicated that the causes of workplace violence stemmed from various factors, including issues within healthcare facilities, the nature of certain clinical settings (like emergency departments, psychiatry, and obstetrics/gynecology), and geographical locations, particularly conflict zones, and other contributing factors included prolonged patient waiting times, unmet patient needs, working night shifts, understaffing during shifts, unsafe work environments (particularly in conflict situations), and the use of substances like khat and alcohol by nurses, other healthcare professionals, patients, or their relatives [7, 15, 20, 21, 25, 26].

### Impacts of workplace violence on the nurse

More than half of nurses changed shifts or rotations [21], Approximately 75% of them expressed concern about violence occurring at work [15], the interaction between a nurse and patient has grown increasingly complex and tense, and allegations have been made regarding the quality of healthcare services provided from admission to discharge [26].

### Availability of policy on violence

Across studies reviewed no existing institutional policy on WPV management was reported [7, 15, 20, 21, 25, 26]. Nurses often underreport incidents of workplace violence due to lack of reporting mechanisms and fear of repercussions, lack of reporting mechanisms and policy frameworks, and lack of awareness about reporting procedures and institutional policies against workplace violence contributes to the availability of such incidents against nurses [7, 15, 21], Lack of administrative action against the perpetrators of the violence even when notified was reported [20]. However, a study done by Weldehawaryat et al. [25] found that 75% of respondents reported the no existence of a procedure for reporting violence in the institution.

## Discussion

This review is the first comprehensive synthesis of workplace violence (WPV) against nurses in Ethiopia, analyzing six studies from various regions—Southern, Northwest, Eastern, and Northeastern Ethiopia [7, 8, 15, 20, 21, 25]. The pooled prevalence of WPV was 39.61%, ranging from 36.52 to 42.70%, indicating an upward trend in violence over the years. These findings are consistent with global studies [28], Pakistan [29], China [28], Ghana [30], and Iran [31] showing that WPV remains a widespread challenge in healthcare settings, particularly among nurses. This is due to their continuous and close interactions with patients and the public [19, 32]. Therefore, recognizing the magnitude of WPV and benchmarking it against global evidence is crucial for informing national strategies, developing robust anti-violence policies, and ensuring a safer work environment for nurses.

The review identified verbal abuse as the most prevalent form of WPV, followed by physical assault, bullying, and sexual harassment [7, 15, 20, 21, 25, 26]. These findings are in line with the study done in Nigeria [33], Turkey [34], Nepal [35], China [35], and India [36, 37]. Such exposure not only undermines nurses’ psychological well-being but also contributes to job dissatisfaction, reduced productivity, and high turnover, affecting healthcare delivery quality. If not adequately managed, such violence may lead to burnout, decreased motivation, and even the departure of nurses from the profession.

Female nurses were found to be disproportionately affected by WPV in three of the reviewed articles [7, 15, 25]. This aligns with a study done in Switzerland [38], Africa [39], and Nigeria [33], which emphasized that gendered power dynamics, societal expectations, and institutional barriers contribute to increased vulnerability among women in the nursing profession. Gender-sensitive workplace policies, confidential reporting mechanisms, and empowerment through training are essential to create a more equitable and secure working environment for female nurses.

Younger nurses under the age of 30 were reported to face higher rates of WPV [7, 15, 25, 40]. This is consistent with studies conducted in Egypt [41], Africa [39], and the USA [12], suggesting that limited professional experience, job insecurity, and poor coping mechanisms contribute to their vulnerability. Given that early-career nurses are still developing their clinical competence and confidence, targeted support, mentorship, and tailored violence prevention training are crucial for their retention and well-being.

Similarly, nurses with fewer years of work experience reported more incidents of WPV [8, 15, 20, 25, 40]. This finding echoes global trends, including those reported in South Africa [42, 43], Taiwan [44], USA [12, 45], where less experienced nurses are more likely to work in high-risk units and lack the assertiveness or knowledge to handle hostile situations. Structured orientation, continuous professional development, and a strong institutional support system can enhance resilience and reduce exposure to violence.

Interestingly, nurses with higher educational levels were also reported to face increased WPV, possibly due to their assignment to critical care or high-pressure units [8, 20, 25, 40], which is similar to studies done in Taiwan [46], Tunisia [47], and the USA [12] settings suggest that educational qualifications may be associated with greater responsibilities, which increases exposure to patient-related frustrations and systemic pressure. Institutions should leverage this group’s knowledge to lead prevention initiatives and policy advocacy while ensuring their safety.

Night shift work was strongly associated with increased WPV [8, 25, 40]. This pattern has been well documented in studies done in the USA [48], Italy [49], and Canada [48]. Interventions such as improved staffing, security protocols, and designated rest areas can mitigate this risk.

Perpetrators of violence ranged from patients and their families to healthcare professionals and supervisors [7, 8, 15, 20, 25, 40], which is similar to sty done in Malaysia [50] and, the USA [51]. Such horizontal and vertical violence has been linked to a toxic work culture, poor conflict resolution strategies, and a lack of administrative accountability [52]. Creating respectful workplace environments and promoting emotional intelligence among all healthcare workers is vital for building a cohesive and safe healthcare team.

A notable association was found between substance use (alcohol and khat) among nurses and increased WPV exposure [7, 25]. Substance use may impair professional judgment and reduce conflict management abilities, a finding supported by reports from the American Hospital Association [53] and studies done in New York [54] and the USA [55]. Promoting substance-free work policies and counseling services can help address this overlooked dimension of WPV.

Short work experience was again emphasized as a key factor in WPV vulnerability as new nurses often lack the confidence and institutional support to navigate aggression from patients [7, 15], which is consistent with studies from Brazil and other developing countries [56]. Structured onboarding programs and fostering a psychologically safe environment are recommended to retain novice nurses.

Nurses working in emergency departments, psychiatric wards, and inpatient services were frequently targeted for violence [8, 15, 20, 25], which is similar to studies done in Africa [57], Nigeria [33], China [58], and Australia [59]. This is due to high-stress situations, frequent patient interactions, complex medical needs, understaffing, high workloads, and lack of security measures. A comprehensive approach involving training, support systems, policy changes, improved communication skills, incident reporting, and collaboration is recommended.

The impacts of WPV are far-reaching. Studies reported that over half of the affected nurses changed their work shifts, and experienced fear, emotional stress, declined job satisfaction, and service quality [15, 20, 21, 26]. This finding is similar to the study done in China [60, 61], USA [12], Spain [62]. Comprehensive policy frameworks, staff well-being programs, and stakeholder engagement can enhance the healthcare system’s responsiveness.

Lastly, the review highlighted an alarming lack of institutional response to reported incidents [7, 15, 20, 21, 25]. This consistent with study finding in Canada [19, 63], China [64], and Jordan [65]. This culture of impunity discourages reporting and perpetuates an unsafe work environment. Leadership accountability, robust legal mechanisms, and visible enforcement of workplace safety policies are essential for change.

### Strength and limitation

This research constitutes the primary comprehensive analysis of workplace violence targeting nurses in Ethiopia, encompassing six scrutinized publications sourced from reputable databases. It underscores the absence of a violence policy despite the elevated incidence of violence against nurses. Urgent consideration is advocated by the research for hospital administrators and policymakers to establish violence prevention protocols for healthcare workers. A notable constraint is the insufficiency of qualitative investigations on workplace violence, potentially obscuring the nuances of violence from the perspective of employees. The limitations of quantitative research methods often hinder a thorough examination of the circumstances, thereby rendering this review an asset for comprehending the issue.

## Conclusion

The prevalence of WPV among Nurses in Ethiopia is increasing in number. Nurses face a high risk of workplace violence due to direct patient contact and the nature of the profession. Thus, healthcare organizations can work towards creating a safer and more supportive environment for nurses, enforce a zero-tolerance policy, improve patient-nurse relationships, and provide psychological support to ultimately reduce the prevalence of workplace violence and ensure the well-being of healthcare professionals.

### Implication to the nurse profession

The issue of workplace violence poses a significant risk to the occupational health and safety of nurses, necessitating immediate intervention from experts in the field of occupational health. Within Ethiopia, there is a noticeable absence of comprehensive policies regarding violence management, underscoring the importance of aligning with global health priorities. Equipping nurses with the necessary skills to identify and address incidents of workplace violence is essential for their readiness and effective response, ultimately safeguarding their well-being and the standard of care they deliver.

### Recommendation for further research

Subsequent research endeavors should incorporate qualitative inquiries aimed at elucidating the characteristics and circumstances surrounding workplace violence encountered by nurses, assessing the efficacy of existing violence prevention measures, and investigating the impact of organizational culture on the prevalence and management of workplace violence.

## Data Availability

The datasets used and/or analyzed during the current study are available from the corresponding author upon reasonable request.
